# Sustainable Prescribing in Secure Services – a Quality Improvement Initiative

**DOI:** 10.1192/bjo.2022.347

**Published:** 2022-06-20

**Authors:** Catherine Weeks, Toral Thomas

**Affiliations:** Norfolk & Suffolk NHS Foundation Trust, Norwich, United Kingdom

## Abstract

**Aims:**

In 2021 The Department of Health published a report into the safer use of medicines in health and justice mental health services, advocating sustainable prescribing as a way of improving patient care and reducing carbon emissions. Improving prescribing behaviour could lead to a reduction of 170,000 kg CO2e per year across England, along with cost savings which contribute to higher value service provision and improved service user experience. We aim to evaluate and improve the prescribing of antipsychotic depot and ‘as required’ (PRN) medication in a male secure unit.

**Methods:**

Baseline data were gathered from the patient population in a male secure unit (1 low and 2 medium secure wards, total 50 beds) in December 2021. This included the number of patients prescribed a depot, the type of depot prescribed and whether or not these were administered at the longest evidence-based interval. As part of a wider trust initiative “prn” medication was moved to a fortnightly review cycle to ensure medication was used for as short a duration as necessary. Over a six-week period medication rationale was analysed and discussed with the Responsible Clinician for the service user to optimise prescribing. Data collected following this intervention was compared with baseline results.

**Results:**

The project found that 26 patients in the service were prescribed an antipsychotic depot in December 2021. In this group 17 (65%) were prescribed their medication at the longest evidence-based interval. Of the 9 (35%) that were not, 5 had clinical reasons why a change would not be appropriate at present, however it was agreed this could be considered later in their pathway. Of the remaining service users, two had their dose of medication reduced and their prescribing interval increased. “As needed” (PRN) medications of 15 patients were evaluated; 9 (60%) had medications prescribed which were not in use (4 patients had 3 or more prescribed not used within 2 weeks). Following intervention this reduced to 2 patients, both of which had only 1 PRN medication which required review.

**Conclusion:**

Deprescribing can have a significant impact on patient care and safety and can reduce the environmental impact of a service. This project demonstrated the advantages gained from regular medication reviews and taking into consideration dose and administration interval when prescribing antipsychotic depots. Using protocols for prescribing as needed medications, a structure for reviewing prescriptions, collaboration with patients and utilising patient group directions where appropriate can all aid in improving prescribing sustainability.

